# Advantages of mini-multileaf in stereotactic radiotherapy

**DOI:** 10.4103/0971-6203.31144

**Published:** 2007

**Authors:** P. G. G. Kurup, V. Murali, A. Sankar

**Affiliations:** Department of Radiotherapy, Apollo Specialty Hospital, Chennai, India

**Keywords:** Dose-volume histograms, mini-multileaf collimator, stereotactic radiosurgery

## Abstract

Over the past few decades, cones of different diameter (12.5 mm to 40 mm) were used for treatment of intracranial lesions. These give very focused dose delivery to the target with minimum dose to outside normal brain tissues. This study is intended to compare the older method of arc-based stereotactic treatments using cones with the new mini-multileaf collimator (mMLC). Treatment plans are made for various sites of intracranial lesions with the cones and mMLC. In case of nonspherical lesions, more than one isocenter is used to get an optimum dose distribution with cones, while a single isocenter is sufficient with mMLC. Treatment plans are compared for irregular lesions using cones with multiple isocenters and mMLC. It is observed that conformity index and dose heterogeneity are better for mMLC based treatments.

## Introduction

**Figure 1:**
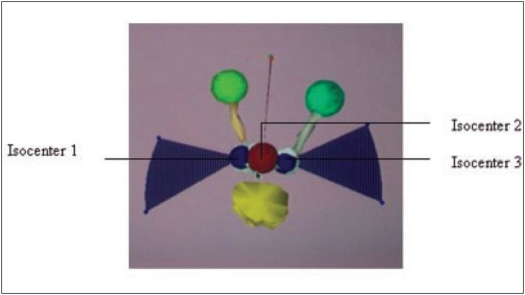
Typical multiple isocenter plan for irregular shaped target

Stereotactic radiotherapy (SRT) is a treatment of choice for small well-defined intracranial lesions. Initially, when SRT was started, only small spherical lesions were considered for these procedures using circular cones fitted to linear accelerators. Very precise gadgets were used for localization of the isocenter during the entire course of treatment.[1] Subsequently nonspherical lesions (elliptical or irregularly shaped) were also considered for stereotactic treatment. Adequate dose uniformity to nonspherical lesions with sparing of normal tissue was possible by using multiple cones and isocenters[2–4] [Figure 1]. There can be two or more isocenters and of varying sizes of cones depending on the shape and size of the lesion to be treated. By this method it is always seen an overlapping region of two cones where the total dose would be 140-200% of the prescribed tumor dose. In this situation the patient has to be set for each isocenter one after the other. The procedure will be more time-bound and may lead to errors in routine set-up for treatment.

By the advent of mini multi leaf collimator (mMLC), the above problems are solved to a great extent.[5] A near circular or elliptical dose distribution also is possible with this kind of mini multileaf shaping, though not entirely circular or elliptical. The biggest advantage is the uniform dose achievement in the target with a single isocenter. Usually, the width of each leaf is around 2.5 mm to 3 mm at the isocenter though it is known that smaller the leaf width, better is the dose conformity, with greater normal tissue sparing.

The dosimetric comparisons are made using circular cones and mMLC, where mMLC has shown to have a marginal advantage in near spherical lesions and considerable benefits in irregularly shaped lesions. In general, while analyzing the dose distribution and dose volume histograms (DVHs), similar results are seen for spherical lesions, whereas mMLC-based radiosurgery spares more normal tissues and results in more uniform dose to the lesions.

There are a few suppliers of mMLC. They are mainly BrainLab GmBH (Germany), Radionics RSA(USA), Direx (UK), Siemens OCS (USA) etc. The maximum size of mMLC shape possible is around 10 cm ×10 cm and the leaf width at isocenter varies from 2.5 to 3.0 mm.

## Materials and Methods

In our hospital, stereotactic procedures started in 1995 with cones supplied by M/s Radionics. Arc-based SRS/SRT treatments were carried out using cones. Irregular lesions were treated with multiple isocenters and spherical / near spherical lesions with single isocenter. Subsequently, mini MLC was procured from BrainLab, which eases the treatment of irregular lesions with a higher conformity.

**Figure 2:**
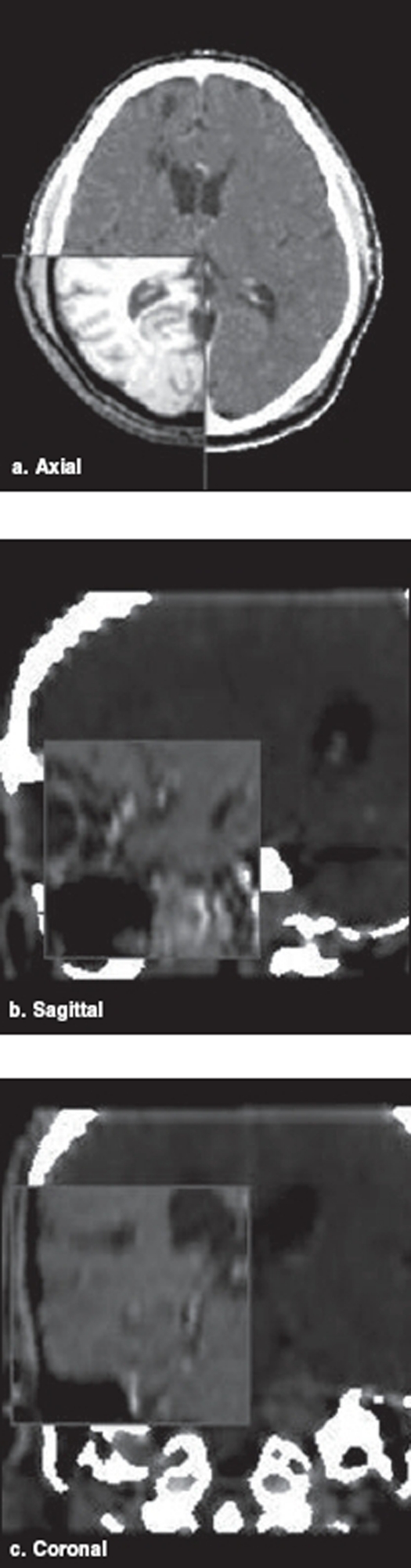
Verification of X-ray CT and MRI image sets along various planes

In stereotactic radiotherapy procedures, CT images are taken as the gold standard for accurate localization purpose. The MRI images may have distortions and hence errors might occur in accuracy. The CT images are normally taken at 2 mm to 3 mm slice thickness and these images are transferred to treatment planning system online. If online facility is not available, transfer through CDs is possible. The MRI is taken routinely and the images are sent to planning system either online or offline. Structures are drawn in both CT and MRI images and are fused by image fusion technique for planning purpose. These images have to be Dicom compatible for use in the planning system. The fusion accuracy of structures in CT and MRI is verified in transverse, coronal and sagittal views with the help of spyglass [Figure 2]. In the event of improper fusion, manual adjustments are made and fused accurately, till a proper correlation is achieved in the anatomical structures of the two sets.

Treatment plans are done with circular arc technique using cones, conformal arc technique using mMLC. The treatment plans are done only for comparative study and not used for patient treatment. Six cases have been chosen for the study. In two cases the target is more spherical in shape and in the remaining four, the target is irregularly shaped. Hence the cases are categorized as spherical and irregularly shaped targets. For the spherical targets, single isocenter is used for planning with cones and mMLC. A margin of 2 mm is added to the spherical targets for the conformal arc plan with mMLC. The cones are chosen in such a way that the aperture size is at least 2 mm bigger than the maximum diameter of the target. For the irregularly shaped targets no margin is added for the circular arc plans with cones or conformal arc plans with mMLC.

### Basis for evaluation of treatment plans

The treatment plans generated with cones and mMLC are evaluated on the basis of conformity index (CI_95_) pertaining to 95% isodose line[6] and dose heterogeneity (DH) within the tumor volume.

Conformity Index is defined as:

${\text{CI}}_{95}=\text{l}+{\text{V}}_{\text{normal tissue}}/{\text{V}}_{\text{ctv}}$

Where V_normal tissue_ and V_ctv_ are the volumes of normal tissue and CTV, respectively comprised within the 95% isodose line.

The DH has been defined as a variation of the dose within the tumor volume, as described by Nezdi *et al*.[7]

$\text{DH}=({\text{D}}_{\text{max}}-{\text{D}}_{\text{min)}}/{\text{D}}_{\text{median}}$

Where D_max_, D_min_ and D_median_ are the maximum, minimum and median dose to the CTV respectively. The DH describes the slope of the cumulative DVH for CTV.

The conformity index and dose homogeneity values for the spherical targets and irregularly shaped targets are shown in Tables 1 and 2.

**Table 1:** Conformity index and dose heterogeneity for spherical targets resulting from different treatment techniques

*Case*	*Parameter*	*Circular arc (single isocenter)*	*Conformal arc using mMLC (single isocenter)*
Case I	CI	1.31	1.25
	DH	0.50	0.16
Case II	CI	1.38	1.28
	DH	0.33	0.17

CI - Conformity index, DH - Dose heterogeneity

**Table 2:** Conformity index and dose heterogeneity for irregularly shaped targets resulting from different treatment techniques

*Case*	*Parameter*	*Circular arc (multiple isocenters)*	*Conformal arc using mMLC (single isocenter)*
Case III	CI	2.13	1.34
	DH	1.04	0.24
Case IV	CI	1.98	1.60
	DH	0.82	0.31
Case V	CI	1.89	1.49
	DH	0.86	0.26
Case VI	CI	1.93	1.42
	DH	0.88	0.36

CI - Conformity index, DH - Dose heterogeneity

## Results and Discussion

**Figure 3:**
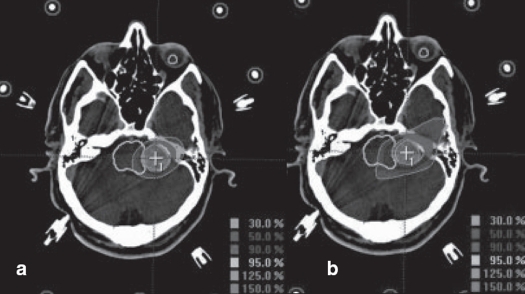
a. Circular arc plan, b. Conformal arc plan. Dose distribution in axial images for spherical target using two different treatment techniques

**Figure 4:**
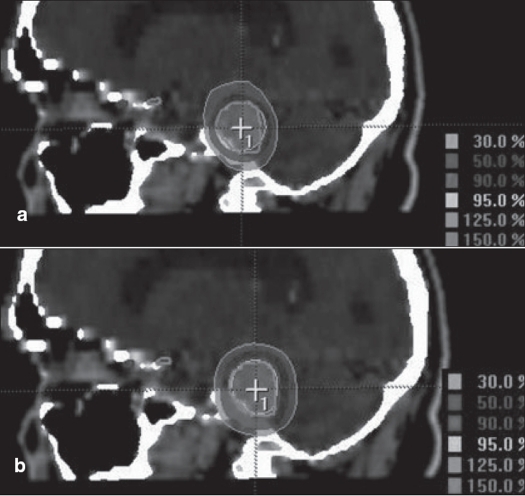
a. Circular arc plan, b. Conformal arc plan. Dose distribution in sagittal images for spherical target using two different treatment techniques

**Figure 5:**
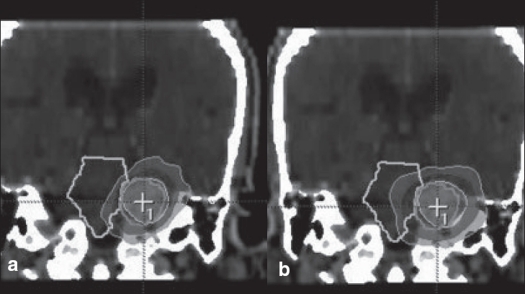
a. Circular arc plan, b. Conformal arc plan. Dose distribution in coronal images for spherical target using two different treatment techniques

**Figure 6:**
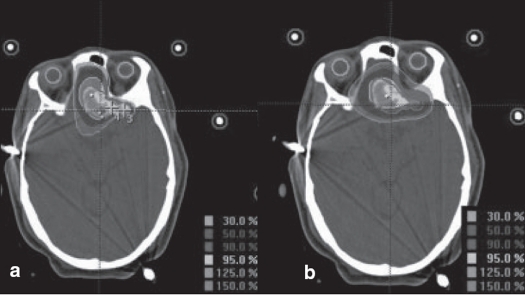
a. Circular arc plan with multiple isocenter, b. Conformal arc plan. Dose distribution in axial images for irregularly shaped target using two different treatment techniques

**Figure 7:**
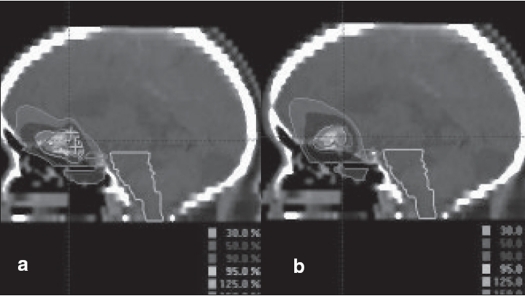
a. Circular arc plan with multiple isocenter, b. Conformal arc plan. Dose distribution in sagittal images for irregularly shaped target using two different treatment techniques

**Figure 8:**
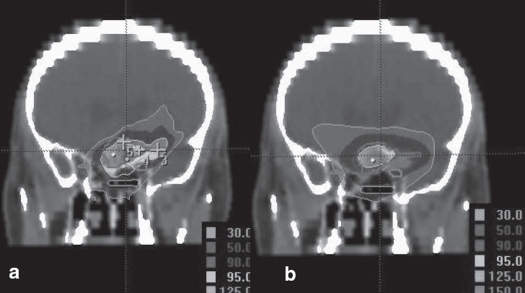
a. Circular arc plan with multiple isocenter, b. Conformal arc plan. Dose distribution in coronal images for irregularly shaped target using two different treatment techniques

We had been using circular collimators from Radionics since 1995 for SRT and radiosurgery. One to five isocenters were used depending upon the shape of lesion. During the past two years, we are treating with mMLC from M/s. BrainLab GmBH, Germany, which has a leaf width of 3 mm. Comparison of dose distribution was made between circular arc plans with cones and conformal arc plans with mMLC. Typical dose distributions in axial, coronal and sagittal planes are shown in Figures 3 to 8 for spherical and irregular-shaped targets.

On analyzing the plans with respect to the target coverage, the CI_95_ for conformal arc plans with mMLC is very much better than the plans with cones. The dose uniformity (calculated in terms of dose heterogeneity, DH) is also much better with conformal arc plans using mMLC. The DH for the plans with cones is roughly about three times that of mMLC plans [Table 2]. In multiple isocenter plans depending on the overlap of the cones and individual isocenter dose, significant volume of the target volume is receiving the higher dose like 110%, 125%, 150% and 175% [Table 3].

**Table 3:** Typical percentage of target volume irradiated by the higher doses in case of multiple isocenter plans for irregular-shaped target volumes

*Case*	*Percentage of volume*			
	
	*110 % dose*	*125% dose*	*150% dose*	*175% dose*
Case III	34.7	19.1	4.5	0.1
Case IV	72.6	48.4	14.5	1.8
Case V	40.2	11.9	2.4	0.1
Case VI	80.0	55.8	19.2	4.3

**Figure 9:**
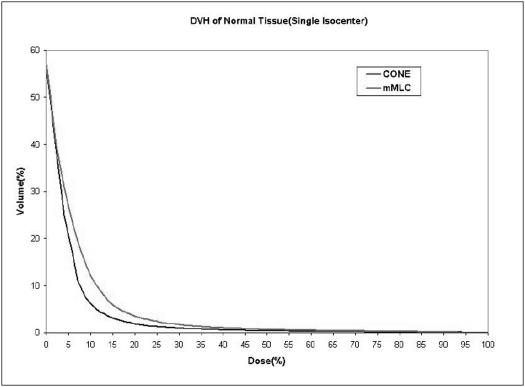
Dose volume histogram of normal tissue for spherical-shaped target using two different treatment techniques

**Figure 10:**
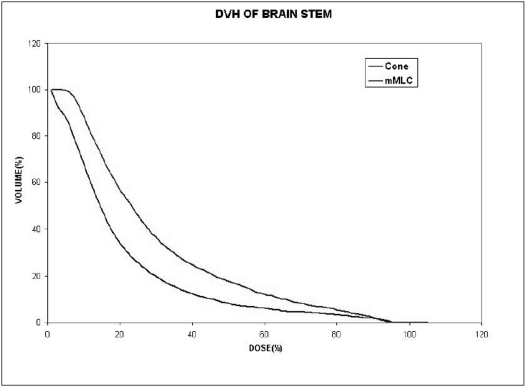
Dose volume histogram of brainstem for spherical-shaped target using two different treatment techniques

When single isocenter is used (for spherical lesions), cone-based radiosurgery spares more normal tissue compared to mMLC-based radiosurgery, especially at high-dose regions as seen in Figs 9 and 10. Typical DVH for spherical lesions by circular arc plan using cone and conformal arc plan using mMLC are shown in Figures 11a and 11b.

**Figure 11a:**
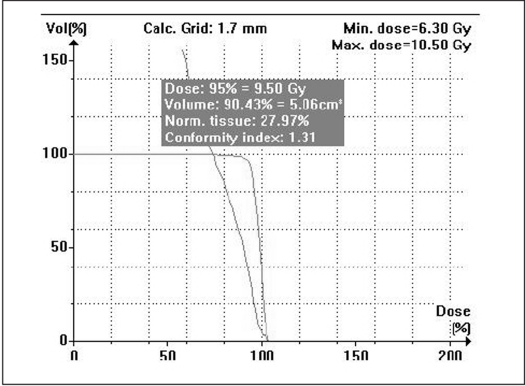
Circular arc plan

**Figure 11b:**
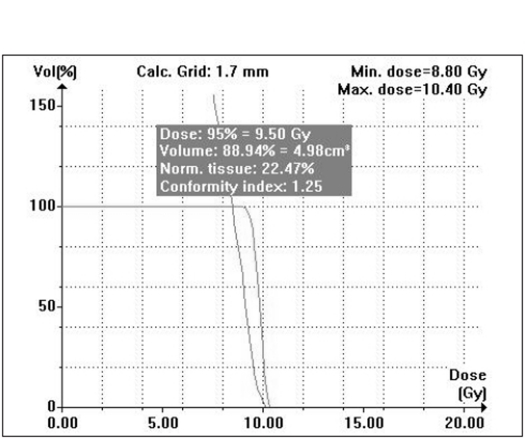
Conformal arc plan: Dose volume histogram of target volume for spherical-shaped target

Compared to the plans using cones with two or more isocenters for irregular lesions, mMLC-based treatment plans give better normal tissue sparing as shown in Figure 12 a and b. The volume of the normal tissue lying within 95% isodose is expressed as the percentage of the total volume of the target. In addition, the dose to critical structures adjacent to the target volume is also less in conformal arc plans, with a more homogeneous dose to the target [Figure 13 a, b, and c].

**Figure 12a:**
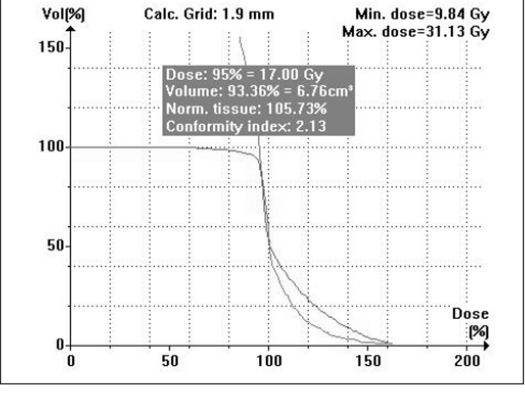
Circular arc plan with multiple isocenter

**Figure 12b:**
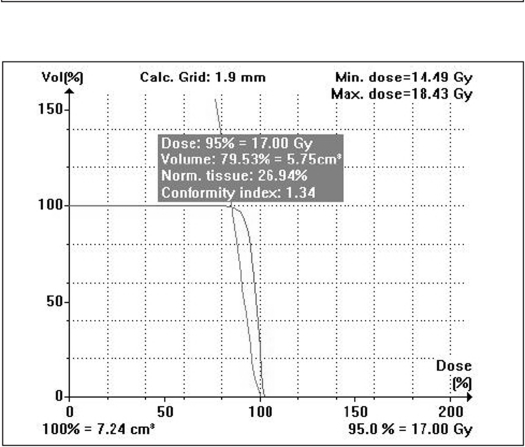
Conformal arc plan: Dose volume histogram of target volume for irregularly shaped target

**Figure 13a:**
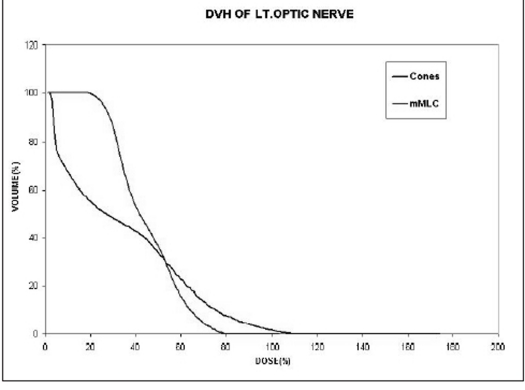
Left optic nerve

**Figure 13b:**
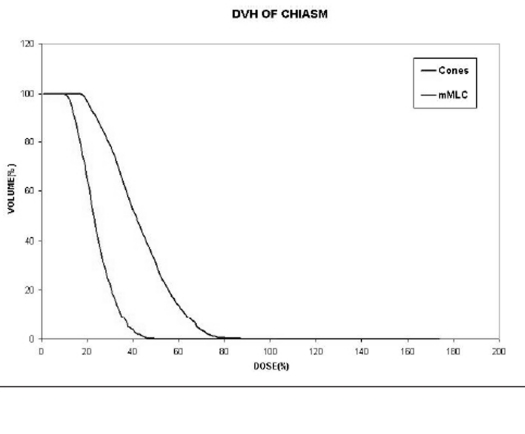
Optic chiasm

**Figure 13c:**
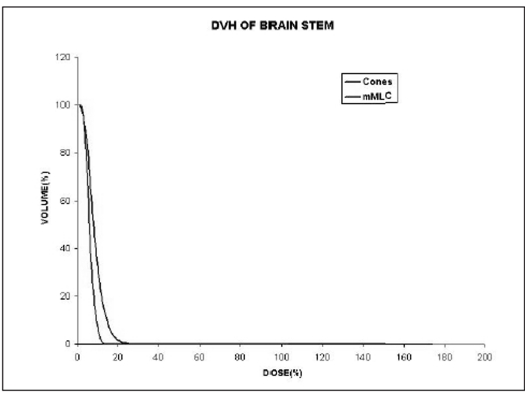
Brainstem: Dose volume histogram of critical structures adjacent to irregularly shaped target using two different techniques

In the case of treatment planning for irregular targets, it takes a much longer time to generate an acceptable plan with cones when multiple isocenters are used. There should not be any overlapping of two arcs of the multiple isocenter. Usually the number of arcs is restricted to the availability of space (between two arcs in this situation) which also makes the dose to normal tissue higher. Moreover set-up time for multiple isocenters consumes a lot of machine hours and man-hours especially for stereotactic radiotherapy.

## Conclusions

Better dose conformity can be achieved for irregular-shaped targets using mMLC-based radiosurgery. Because of better conformity the dose to normal tissues is less in mMLC-based plans compared to cone-based multiple isocenter radiosurgery. The multiple isocenter technique used in cone-based radiosurgery results in a high degree of dose heterogeneity in the overlapping regions. DH is better in mMLC-based radiosurgery even for highly irregular-shaped targets.
